# Four Cases of Tubercular Meningitis

**Published:** 1881-08

**Authors:** Charles K. Mills, Lambert Ott


### FOUR CASES OF TUBERCULAR MENINGITIS.

BY CHARLES K. MILLS, M.D., AND LAMBERT OTT, M.D.

The cases here reported occurred in the practice of Drs. M. Franklin and L. Ott, and were seen by Dr. Mills in consultation. The autopsies were made conjointly by Drs. Franklin, Ott and Mills, and the microsopical examinations by Dr. L. B. Hall.

Case I.—G., female, five years of age, had a good family history, her parents being quite healthy, and her grandparents having reached a good old age: no consumption had ever been known in the family. The child had never been as bright and active as other children of her age; she seemed to like solitude. For two weeks she had been peevish and fretful; not playing, refusing to eat, and appearing unconcerned when addressed. Her parents noticed a tendency to unnatural drowsiness. She was brought to Dr. Franklin's office, and while there went to sleep, at the same time vomiting. On the way home, however, she walked as well as a healthy child. The next day she was seen at home; she did not stir when approached, but opened her eyes, stared and relapsed into a drowsy state. Her pulse was 96, irregular and soft; temperature 98.1°. Respirations was 27, and presented striking irregularities, the *irregularities* occurring in nearly *regular* order. Two to four sighs took place in the course of a minute. A very long sigh would be drawn, followed by a cessation of respiration, lasting ususally from ten to fifteen seconds, sometimes being shorter. Short and rapid breathing then occurred, increasing in length with each inspiration until it reached about a normal rate, continuing at this for three or four respirations, when there would be another sigh, and then another pause. This would be again followed by scarcely perceptible breathing, but gradually increasing as before. This peculiar "ascending" respiration did not follow every sigh. Supposing that four sighs occurred in the minute, two or three would be followed by the hiatus and gradually increasing respiration, while the other one or two, much shorter, would be followed by ordinary respiration. Lifting the child, pricking her with a pin, or otherwise irritating her, would disturb the peculiar irregular rythm of the respiration. When touched or moved she cried out vehemently, at the same time manifesting great irritability, but as soon as the disturbance ceased she immediately fell asleep. When irritated her face became intensely flushed. *Tache cerebrale* was well marked, the red streak coming slowly when the finger was drawn over the surface, and then gradually fading. The pupils were dilated, responding feebly to light. The skin was dry and scaly, the extremities were cold. The urine was passed in bed; the bowels were constipated. The belly was not retracted. She was considerably emaciated. Physical examination of the lungs showed dullness over the left apex, with harsh respiration. On the day on which these notes were made, about the fourteenth since anything wrong had been observed, she had a slight spasm.

15th day. Her pulse was 96, rectal temperature 97°, respirations 20. At every fifth and seventh respiration was a sigh. The pupils were unequally dilated. She was continually drowsy, half awaking now and then, and staring vacantly. She threw off all covering day and night, seeming to wish to be naked. The abdomen was beginning to be retracted. Her bowels were opened, the passage being hard. The extremities were warmer than on the previous day. She was given a piece of solid food, a favorite dish, which she bit eagerly and chewed a few seconds, and then fell off to sleep with the bolus in her mouth. She was much more irritable than on the previous days. When both thighs were seized and the femur squeezed, she would give a piercing scream. This was a striking peculiarity. Compressing other parts of the extremities did not elicit any cry.

On the 16th day her condition was very much the same as on the previous day; pulse 84, temperature 98.6°, respirations 24. On auscultating the chest, a peculiar alteration in the normal condition of the respiratory sounds was observed. Sometimes for three or four respirations the inspiratory sound would be prolonged much beyond the expiratory, and this was followed alternately by a prolonged expiratory sound. The end of the stethoscope left a white ring with a red centre, and shortly after removing it, the entire ring would become red. The face had a permanent flush, which was aggravated when the child was disturbed. The abdomen was much retracted.

On the 17th day the pulse was 124, temperature 99.2°, respirations 20. The inspiratory sound was much prolonged. She could not stand unsupported. The nose and left cheek showed a bright flush; the left side and extremity were deeply flushed.

18th day. Pulse 120, temperature 99.5, respirations 24. Her condition about the same, only the skin was quite dry.

19th day. Pulse 118, temperature 97.2°, respirations 24. She had an involuntary passage from the bowels, in bed. The muscles of the back and neck were stiff, with a little inclination in opisthotonos. The knee joint was very stiff; other joints were normal. The eyelids twitched convulsively. She was still very stupid, lying quiet, with an expressionless countenance, but showing great irritability, with an instantaneous flush of the face, when disturbed, Her cough continued to increase. When the femurs were compressed she quickly thrust out her hand to resent the annoyance, crying as if in pain, The same handling of other parts of the extremities gave apparently no pain.

No notes were made on the 20th day.

21st day. Pulse 128, temperature 99°, respirations 24. The abdomen was much retracted. When a rapid motion was made towards the open eyes she rapidly closed them, as if in dread of a blow.

22d day. Pulse 134, temperature 99°, respirations 40. The respirations still showed the irregularities already described.

23d day. Pulse 120, temperature 97.5°, respirations 16. She snored considerably. While watching her, the face suddenly became intensely flushed, without any external disturbance. She had ptosis of the left uppei lid, and partial closure of the right upper lid during sleep. Loss of power in the right arm and leg became manifest. Sensation seemed in no way altered. Examination of the urine showed that neither albumen nor sugar were present.

24th day. Pulse 128, temperature 98°, respirations 24.

25th day. Pulse 125, temperature 98°, respirations 28. A swollen, tympanitic belly was taking the place of the retracted abdomen.

26th day. Pulse 144, temperature 99.7° respirations 40.

27th day. Pulse 160, temperature 98.8°, respirations 20. She threw her head back with every inspiration, at the same time opening her mouth as if unable to obtain sufficient breath. Every few minutes the face would suddenly become flushed and would so remain for about three minutes.

On the 28th day she died, while trying to take a little milk, which she was unable to swallow.

*Autopsy.*—The central base from optic chiasm to medulla oblongata, showed advanced purulent exudation, involving the optic nerves and tracks, and the third and fourth nerves. The latter nerves, particularly on the left side, were bound down. Tubercles were found along the vessels far out in the Sylvian fissures. A few miliary tubercles were found in the velum interpostium. A large amount of fluid was found in the ventricles.

Case 2.—Male infant, ten months old. The father had died from lung disease of a few months' duration. The babe had thrived well on artificial nourishment until three weeks before he came under medical care, when he was taken with vomiting and purging, passing blood and mucus; this lasted two days, and he was afterwards cross and peevish. When first examined he had slight cough, was irritable, continually moaning, not satisfied in any position. He had great thirst. His bowels were opened twice daily. Saliva dribbled from his mouth. His physiognomy presented a peculiarity, as follows: The longitudinal and transeverse wrinkles of the face successively appeared and disappeared, the eyelids opening and closing, at the same time throwing his head back every few moments, as if in a fit of anger. The temperature was 102°. His condition remained nearly the same for about a week. About two and a half weeks from the beginning of the illness he fell into a stupor, only awaking to take a little milk when placed in his mouth, and then showing great irritability.

On the 19th day his pulse was 140, temperature 102°, respirations 40. His skin was very dry. He moaned a great deal during sleep, and when disturbed manifested extreme irritability.

On the 20th day he was still sleeping, and when awakened stared with a frightened expression. He was usually quite pale, but sometimes a flush would suddenly appear. His abdomen was beginning to be a little retracted. Several times during the day he perspired considerably. His bowels were opened three times. Pulse was 120, temperature 102°, respirations varied from 10 to 20 per minute. On watching the breathing, and judging merely by sight, it was noticed that two respirations occurred, then a sigh followed by a pause, or, at least, no perceptible movement, for from twenty to twenty-five seconds; after this came two rapid respirations. On pressing the hand deeply into the abdomen, however, during this apparent pause of twenty-five seconds, rapid and shallow breathing was discovered.

On the 21st day the pupils were sensitive to light. The bowels were opened once. The neck and back were quite rigid, inclining to opisthotonos. The retraction of the abdomen was increasing. Tache cerebrale was manifest.

22d day. Flushes occurred much oftener during the evening. He perspired profusely. He whined when much pressure was exerted on the thigh. Pulse 120, temperature 101.2°, respirations 56.

23d day. A peculiar odor was given off by the patient, like that of the perverted secretions of cutaneous glands. Washing did not cause the odor to disappear. The patient screamed suddenly at intervals, and had convulsive twitchings of the hands and feet, lasting for an hour. The eyelids also twitched at times. The pulse was fluttering and could not be counted. Temperature 103°, respirations 48.

24th day. For twenty minutes at a time he tossed his head from side to side, and then remained quiet for about the same period, when the movements recurred. He had now also rapid oscillations of the eyes, with contracted pupils. Sensibility was abolished in the left arm and both lower extremities. Temperature 107°; respirations 64, regular and labored. He died on the evening of the 24th day.

*Autopsy.*—The anterior fontanelle was very little closed, considering the patient's age. The dura mater of the convexity was adherent to the skull. The tentorium, on its cerebellar aspect, showed some redness and exudation. The pia mater of the under surface of the temporo sphenoidal lobes was hyperæmic. The pia mater covering the orbital surfaces of the frontal lobes was somewhat opaque, and showed numerous miliary tubercles, more on the left than on the right side. The membrane covering the entire central portion of the base, from a point one-half inch in front of the optic chiasm backward to the pons, was milky white and thickened. The chiasm was bound down with exudation. The Sylvian fissures were glued together, exudations and deposits being found along the Sylvian vessels. Scattered tubercles were found over the pia mater of the convexity of both frontal lobes. The posterior horns of the ventricles were much dilated.

*Microscopical Examination by Dr. Hall.*—The membranes were thickened by an increase of fibrous tissue. There was an infiltration of rounded cells, chiefly along the vessels, forming a wedge-shaped ring on the small arterioles, where they dip into the cortex. On the vessels of the pia mater they formed oval-shaped masses, not encroaching on the calibre of the tube. These, as well as the thickened membrane, took the stain more readily than the rest, showing their active condition.

Case 3.—M., male, aged two years and six months, had always enjoyed good general health, but had passed through a severe attack of summer complaint four months previous to coming under observation. His father died of consumption. Nine days before he was seen professionally he was attacked with apparently causeless vomiting, with some diarrhœa, fever and general irritability. The next day the mother noticed that the child had a fixed and staring expression. He remained restless, irritable and out of sorts, but without any change of note in his condition for seven days, when one of us was called in to see him. His eyes were fixed and staring. It was impossible to attract his attention. Even screaming in his ears had no effect. His pulse was 108, temperature 101.4°, respirations 30. Respiration was irregular and peculiar; five to six breathings would be succeeded by a sigh, and this would be followed by a pause of fifteen to twenty seconds. Faint respiratory movements could not be detected when the hand was pressed deeply into the abdomen. After the pause, a few rapid, short respirations—but each increasing in length—

were taken, until easy, regular breathing was reached. This would be followed again by a sigh, pause and ascending respiration. The left pupil was dilated. He had a cough, apparently due to bronchial catarrh. The abdomen was a little flaccid and somewhat retracted.

We will not give the record of daily observations in this case, the condition not changing much, and not differing markedly from that noted in other cases.

The following notes were made on the 20th day: pulse 160, soft and irregular; temperature 104.2°; respirations 68, labored and different from preceding days. He would have five rapid and lengthy inspirations, with an expiratory moan; then ten rapid, short and nearly imperceptible inspirations, not attended with the expiratory moan; these would again be followed by the lengthy inspirations and expiratory moan. The pupils were equal, the eyes staring and lids still wide open. The nostrils were constantly dilating. Mucous rales were present in the chest, but percussion showed no dullness. The cheeks were flushed. Tache cerebrale was quite marked. He swallowed with difficulty. The skin was moist. The tongue was coated, white and dry. The bowels were constipated, urine passed in bed. Tightly grasping the thighs did not seem to cause pain. The extremities were in a state of tonic spasm, now and then alternating with clonic spasms. The patient died on the 21st day of the disease.

*Autopsy.*—The pia mater over both orbital regions was deeply congested. Thickening of the membrane and purulent exudation were present over wide-spread regions of the base of the brain. Beginning one-half inch in front of the optic commissure the exudation extended out along both Sylvian fissures—on the left as far as the ascending convolutions, on the right for a distance of about one inch; it was present over the anterior ends of both temporo sphenoidal lobes, and over the central portion of the base from the optic commissure to the pons. The olfactory, optic and oculo-motor nerves were slightly bound down. Over the anterior arachnoid space the membrane was of a deep red, but less opaque than in the other regions described. The pia mater over the posterior part of the first frontal convolution of the right side was slightly opaque and thickened. The ventricles were filled with fluid.

*Microscopical Examination by Dr. Hall.*—Thickened membrane and infiltration of rounded cells along the vessels were found here also, the membrane and the masses formed taking the stain more readily than other parts.

Case 4.—J. H., male, aged 6 years. The family had a tubercular history, and one patient died of brain trouble.

For two weeks the boy had been vomiting before breakfast, and had been complaining of pain in the abdominal region. He did not wish to go to school, and when compelled to do so by his parents, returned feeling quite sick, One morning Dr. Ott happened in the house, non-professionally, and saw him, lively and looking well. Shortly afterward the patient went out with his sled, and after being out twenty minutes his companions noticed that he had a peculiar stiff appearance. Calling to him and receiving no response, they took hold of him and found him quite rigid. He was carried home. He was quite insensible. He was placed in a warm bath and cold applied to his head, but without effect.

One hour after the beginning of the seizure he presented the following condition: his eyes were staring, now and then slowly oscillating, the pupils were dilated, the lids were twitching. The muscles of the mouth also twitched constantly, and a stream of frothy saliva also flowed from the left corner of the mouth. His breathing was very irregular, jerking, and occasionally sighing or catching; sometimes expiration was attended with a moan. He was very pale, his lips blue. His pulse was 160. The extremities were cold and uninvolved in the spasm. Chloroform controlled the convulsions for the time being, when the lids closed, the pupils contracted and the pulse fell to 140. In half an hour he again opened his eyes, the lids began twitching, the eyeballs suddenly oscillating, but now and then becoming fixed. Three minutes after these prodromic spasms the muscles of the face became involved in the twitching; a flow of saliva followed, and then came a general convulsion. Being unable to swallow, he was given an enema of potassium bromide and chloral, and chloroform was administered by inhalation. This treatment eventually subdued the spasms, and and the patient relapsed into a comatose condition. The corneæ were insensible, the pupils contracted, and the pulse very weak. Three hours later another convulsion came on, beginning with oscillation of the eyes and twitching of the lids, and being soon followed by a clonic spasm of the left arm and leg. A hypodermic injection of morphia quieted him in ten minutes. His breathing became stertorous, his face livid, bronchial and tracheal rales were present, his pulse was very weak and frequent, and he showed every sign of impending death. He was turned on his side, to rid his chest of mucus and ease his breathing. Stimulants with potassium bromide and chloral were ordered.

Remaining in this condition about six hours, his mother called to him, when he opened his eyes and seemed bewildered. He was given drinks, which he swallowed, the first time since the beginning of the attack. He spoke and appeared quite rational. His bowels were now opened by means of a hydragogue cathartic. From this time until morning, being about eight hours, he alternated between spells of dozing and awaking, but seeming always confused.

In the morning he was quite irritable. He protruded his tongue when ordered; it was covered with a white fur, was moist and not bitten. Vomiting now set in. Temperature 101° F., pulse 144, soft and compressible, respirations 42, irregular, sometimes sighing, imperceptible at times, and then gradually lengthening and becoming stronger. The pupils were contracted and sensitive to light. On pricking his face and upper extremities, even in drawing of blood, he did not wince. Sensation in the lower extremities, however, was much better. The abdomen was very much retracted. Motion was good in all parts of the body, especially observed while throwing himself about on the bed. He articulated well. During the remainder of this day he was in a semi-stupor. In the evening he showed some improvement, sleeping more naturally and requesting drinks. His pulse was 104, temperature 101° F., respirations 36 and quite irregular. He vomited occasionally. The pupils were contracted and acted sluggishly. He was ordered ice to the head and counter irritation to the back of the neck.

On the third day he was very restless, and had been so during the previous night. He had some cough. His abdomen was more retracted. His pulse was very frequent, temperature 100.4° F., respirations 36. He vomited and complained of headache. During the entire day he was drowsy. His skin was more sensitive. He lay with his eyes partially opened, staring vacantly. On looking directly into his face he neither moved nor closed his eyelids. The pupils were dilated and sensitive to light. In the evening the pulse was 144, soft and irregular, now and then bounding; temperature 101.7°, and respirations 32, regular but labored. His urine was found to be acid in reaction, and contained neither albumen nor sugar.

On the morning of the fourth day the pulse was 150, feeble, temperature 100.4°, respirations 28. Bronchial rales were present, with considerable cough. He was indifferent to surroundings; when spoken to did not answer. In the evening pulse was 150, temperature 102.2°, respirations 28. He passed his urine in bed. His skin was moist. The right pupil was dilated, the left was about normal. The *tache cerebrale* was very distinct. His respirations were regular and labored for three or four minutes, then came a short but distinct pause, lasting from one-fourth to one-half a minute, and followed by coughing. With every inspiration his lids twitched. He was now much emaciated.

On the fifth day the staring was increased. When the eyes had been closed for some time and the lids were suddenly raised, a contraction and dilatation of the pupils, lasting thirty seconds, would occur. The pulse was 180, temperature 103.5°, respirations 40, very labored and abdominal, tonge dry and coated. The vomiting had ceased, the bowels were constipated. Flushes suddenly appeared, lasting a few minutes, and replaced by a pale, waxy appearance. *Tache cerebrale* was still quite marked. He swallowed with difficulty- In the afternoon his pulse became quite imperceptible, the temperature 101.5°, and breathing quite labored. Spasms occurred, involving first the left side of the face, and afterwards the right side, and then both upper extremities. They lasted half an hour, and in another half hour he was dead. For .eight hours before death he did not voluntarily move his left arm.

*Autopsy.*—No opacity or exudation was anywhere discoverable, but the pia mater, both of the base and convexity, was streaked with red and more adherent than usual. The opacity, exudation, and thickening found in the central regions of the base in the other cases, were entirely absent in this case. A few miliary tubercles, or what appeared to be miliary tubercles, could be made out by the naked eye, in the pia mater of the anterior frontal portion of the convexity, particularly in the left hemisphere. The specimen examined microscopically by Dr. Hall was taken from this region. No hydrocephalus was present. Coagulated blood was found in some of the vessels of the base. The floor of the fourth ventricle, on each side of the median line, both above and below the auditory tracts, was deeply conjested

*Microscopical Examination by Dr. Hall.*—The membranes were thickened, and stained more deeply than normal. Here and there along the vessels was a pyriform swelling. No infiltration along the arterioles, where they dipped into the cortex, could be found. The same lesion seemed to be present here as in the other two cases, but in an earlier stage.

*Remarks.*—We shall give these cases without extended remarks. Most of the symptoms, physical and psychical, were those usually to be expected in cases of tubercular memingitis The great pain produced on squeezing the femurs, in Case 1, was a peculiar manifestation, and one to which we do not remember ever having known attention to be called.

Cheyne-Stokes respiration was observed in three of these cases. Even in the fourth the breathing was peculiar. It will only be necessary to recall that in this form of respiration brief periods of apnœa recur at intervals. Prof. Wm.

Pepper, in the Philadelphia *Medical Times* for May 27, 1876, published an article entitled *Remarks on Cheyne Stokes Respiration, especially in connection with Tubercular Meningitis.* He gives the notes of two cases, and reference is made to reports of cases by other authorities. We are certainly inclined, with Dr. Pepper, from our own experience in the four cases here reported, to attribute to Cheyne-Stokes respiration positive diagnostic value in tubercular meningitis.

In Case 1 the temperature ranged between 97° F. and 99.6° F.; in case 2 fron 101.2° to 107° just before death ; in Case 3 from 101.4° to 104.2°, and in Case 4 from 101° to 103°. Observations on temperature in tubercular meningitis show, as is well known, great irregularities. One of the most recent investigations is that of Jules Turin. (*Jahrbuch fur Kinderheilkunde*, vol. xvi). Our observations in these cases agree with some of the conclusions of Turin, but differ from others. The first three cases of Turin are as follows: 1. Tubercular meningitis is always accompanied by a rise in temperature in one or the other of its stages, but very seldom during its entire duration. 2. In a few cases only does the disease begin with a sudden rise of the bodily heat, as in some forms of acute disease. 3. The thermometric results are extraordinarily variable, so that it is quite impossible to establish any typical temperature curve. With these conclusions our observations are particularly in accord. His fourth conclusion is that in uncomplicated cases of tubercular meningitis the temperature rarely exceeds 102.2° F., and generally varies between 100° and 102°, and in many sink some degrees below the normal. In the first of our cases the temperature remained a little below or a little above the normal all the time. In all of the other cases it reached a higher point than 102° before death, in Case 2 even reaching 107°.

Case 4 differed from the other three cases in several particulars. A cataleptoid condition and severe spasms ushered in the attack, although he had vomited several times and complained of pain in the abdomen. The patient died within a week after the occurrence of the first spasm. Vomiting, headache, elevated temperature, irregular respiration, but not of the Cheyne-Stokes type, alterations in the pupils, *tache cerebrale*, were among the symptoms. Nystagmus was a symptom worthy of special note. The case was probably one in which miliary tubercles in small quantities were here and there in the entire pia, although only visible to the naked eye in the antero-frontal regions of the convexity. The tubercular inflammation was apparently more intense at the convexity than at the base. It is not impossible that the nystagmus, and local and general convulsions, were due to direct irritation, by an inflammatory process, of the motor regions of the cerebral cortex. This case should teach us not to make the mistake of supposing that tubercular meningitis is not present because we do not have exudation and suppuration of either base or convexity. The minute tubercles may only be discovered at particular regions when the inner surface of the pia mater is very carefully examined. It may, indeed, be necessary to resort to the microscope to set any doubts at rest. Miliary tubercles may exist in large numbers without causing purulent inflammation of the pia anywhere.

